# (−)-Norfluoro­curarine ethanol monosolvate

**DOI:** 10.1107/S1600536813015365

**Published:** 2013-06-15

**Authors:** Shahobiddin M. Adizov, Jamshid Ashurov, Zokir Karimov, Pattax Kh. Yuldashev, Bakhodir Tashkhodjaev

**Affiliations:** a S.Yunusov Institute of the Chemistry of Plant Substances, Academy of Sciences of Uzbekistan, Mirzo Ulugbek Str., 77, Tashkent 100170, Uzbekistan; b Institute of Bioorganic Chemistry, Academy of Sciences of Uzbekistan, Mirzo Ulugbek Str., 83, Tashkent 100125, Uzbekistan; c Tashkent Institute of Irrigation and Melioration, Kori-Niyoziy Str., 39, Tashkent, 100000, Uzbekistan

## Abstract

The title compound, C_19_H_20_N_2_O·C_2_H_5_OH, is an ethanol solvate of an indol alkaloid which was extracted from the plant *Vinca erecta*. The fused piperidine ring adopts an approximate boat conformation and the pyrrolidine ring an envelope conformation with one of the methyl­ene C atoms at the flap. An intra­molecular N—H⋯O hydrogen bond forms an *S*6 ring motif. In the crystal, norfulorocurarine and ethanol mol­ecules are linked into a chain along the *c-*axis direction through N—H⋯O and O—H⋯N hydrogen bonds.

## Related literature

For the biological activity of plants containing norfluoro­curarine class alkaloids, see: Lavrenova & Lavrenov (1997 ▶). For the isolation of norfluoro­curarine from the plant *Vinca erecta*, see: Yunusov & Yuldashev (1952 ▶, 1957 ▶). For the physical properties and crystal structures of several norfluoro­curarine solvates, see: Tashkhodjaev *et al.* (2011 ▶).
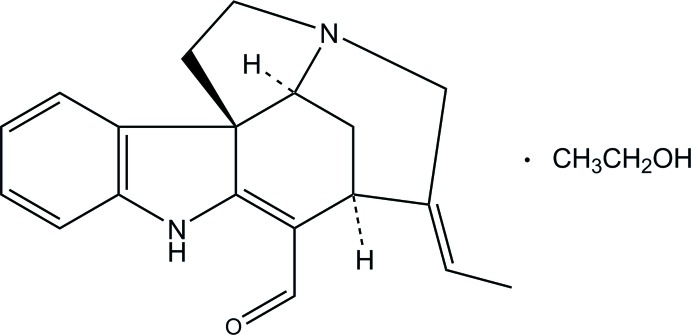


## Experimental

### 

#### Crystal data

C_19_H_20_N_2_O·C_2_H_6_O
*M* *_r_* = 338.44
Orthorhombic, 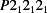

*a* = 7.0138 (5) Å
*b* = 16.090 (1) Å
*c* = 16.490 (2) Å
*V* = 1860.9 (3) Å^3^
*Z* = 4
Cu *K*α radiation
μ = 0.62 mm^−1^
*T* = 293 K
0.60 × 0.30 × 0.20 mm

#### Data collection

Oxford Xcalibur, Ruby diffractometer
Absorption correction: multi-scan (*CrysAlis PRO*; Oxford Diffraction, 2009 ▶) *T*_min_ = 0.801, *T*_max_ = 0.884
5608 measured reflections
3178 independent reflections
2393 reflections with *I* > 2σ(*I*)
*R*_int_ = 0.027

#### Refinement

*R*[*F* ^2^ > 2σ(*F* ^2^)] = 0.051
*wR*(*F* ^2^) = 0.142
*S* = 0.99
3178 reflections
237 parameters
H atoms treated by a mixture of independent and constrained refinement
Δρ_max_ = 0.33 e Å^−3^
Δρ_min_ = −0.19 e Å^−3^

### 

Data collection: *CrysAlis PRO* (Oxford Diffraction, 2009 ▶); cell refinement: *CrysAlis PRO*; data reduction: *CrysAlis PRO*; program(s) used to solve structure: *SHELXS97* (Sheldrick, 2008 ▶); program(s) used to refine structure: *SHELXS97* (Sheldrick, 2008 ▶); molecular graphics: *SHELXTL* (Sheldrick, 2008 ▶); software used to prepare material for publication: *SHELXTL*.

## Supplementary Material

Crystal structure: contains datablock(s) I, GLOBAL. DOI: 10.1107/S1600536813015365/is5277sup1.cif

Structure factors: contains datablock(s) I. DOI: 10.1107/S1600536813015365/is5277Isup2.hkl

Additional supplementary materials:  crystallographic information; 3D view; checkCIF report

## Figures and Tables

**Table 1:** Hydrogen-bond geometry (Å, °)

*D*—H⋯*A*	*D*—H	H⋯*A*	*D*⋯*A*	*D*—H⋯*A*
N1—H1⋯O1	0.87 (3)	2.31 (3)	2.785 (4)	115 (3)
N1—H1⋯O2	0.87 (3)	2.17 (3)	2.961 (4)	152 (3)
O2—H2⋯N4^i^	0.96 (5)	1.85 (5)	2.805 (4)	172 (5)

Symmetry code: (i) 
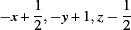
.

**Fig. 1.**
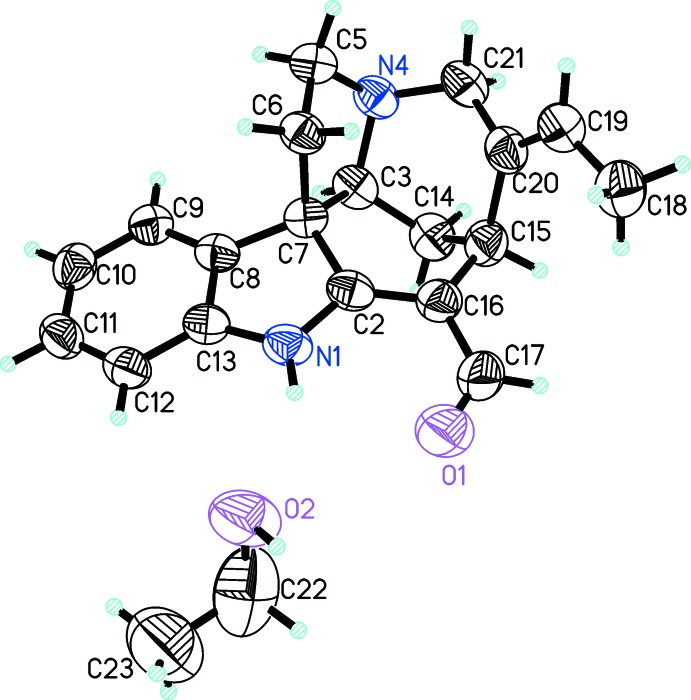
The molecular structure of the title compound, showing the atomic numbering scheme and displacement ellipsoids drawn at the 30% probability level.

## supplementary crystallographic information

### Comment

Because of possessing high biological activity, plants containing norfluorocurarine class alkaloids have been widely used in traditional medicine. A number of plants as *Vinca major L.* and *Vinca herbacea Waldst. et Kit.* are examples which are used as a healing agent in traditional medicine (Lavrenova *et al.*, 1997). Norfluorocurarine for the first time was extracted from the root of *Vinca erecta* and called vincanine (Yunusov & Yuldashev, 1952). Later the alkaloid was extraxted from the upper parts of the same plant (Yunusov & Yuldashev, 1957).

Earlier unsolvated crystal form of (-)-norfluorocurarine was obtained from acetone and stereochemistry studied by Tashkhodjaev *et al.* (2011). When we used ethanol as a solvent, XRD experiments showed the solvated structure in the molar ratio 1:1. Crystals which were obtained in acetone and in ethanol showed the same absolute configuration of alkaloid molecule. But the crystal packing and intermolecular bonds are quite different.

In the molecule, the carbonyl group is oriented to N1—H group. The torsion angle of C2═C16—C17═O1 atoms is -11.7 (5)°. Therefore, the carbonyl group and N1—H group form an intramolecular hydrogen bond N1—H···O1═ C17 (Table 1). In the crystal, the hydroxyl group of solvated ethanol molecules forms intermolecular hydrogen bonds with norfluorocurarine N1 and N4 atoms (Table 1). The hydrogen bonds links the norfluorocurarine and ethanol molecules along the *c* axis.

### Experimental

The title compound was isolated from the chloroform fraction of the plant *Vinca erecta* by a known method (Yunusov *et al.*, 1957). Norflurocurarine was dissolved in ethanol and evaporated in room temperature and obtained suitable for X-ray crystals. Since the crystal was unstable in air, we covered it with epoxide glue.

### Refinement

The H atoms bonded to N1 and O2 were located in a difference Fourier map and refined isotropically. The H atoms bonded to C atoms were placed geometrically (with C—H distances of 0.98 Å for CH, 0.97 Å for CH_2_, 0.96 Å for CH_3_ and 0.93 Å for C_ar_) and included in the refinement in a riding motion approximation with *U*_iso_(H) = 1.2*U*_eq_(C) [*U*_iso_(H) = 1.5*U*_eq_(C) for methyl H atoms].

### Crystal data

**Table:**

C_19_H_20_N_2_O·C_2_H_6_O	*F*(000) = 728
*M**_r_* = 338.44	*D*_x_ = 1.208 Mg m^−^^3^
Orthorhombic, *P*2_1_2_1_2_1_	Cu *K*α radiation, λ = 1.54184 Å
Hall symbol: P 2ac 2ab	Cell parameters from 1783 reflections
*a* = 7.0138 (5) Å	θ = 3.8–75.7°
*b* = 16.090 (1) Å	µ = 0.62 mm^−^^1^
*c* = 16.490 (2) Å	*T* = 293 K
*V* = 1860.9 (3) Å^3^	Prysmatic, orange
*Z* = 4	0.60 × 0.30 × 0.20 mm

### Data collection

**Table:**

Oxford Xcalibur, Ruby diffractometer	3178 independent reflections
Radiation source: fine-focus sealed tube	2393 reflections with *I* > 2σ(*I*)
Graphite monochromator	*R*_int_ = 0.027
Detector resolution: 10.2576 pixels mm^-1^	θ_max_ = 70.0°, θ_min_ = 3.8°
ω–scan	*h* = −8→7
Absorption correction: multi-scan (*CrysAlis PRO*; Oxford Diffraction, 2009)	*k* = −19→17
*T*_min_ = 0.801, *T*_max_ = 0.884	*l* = −18→20
5608 measured reflections	

### Refinement

**Table:**

Refinement on *F*^2^	Secondary atom site location: difference Fourier map
Least-squares matrix: full	Hydrogen site location: inferred from neighbouring sites
*R*[*F*^2^ > 2σ(*F*^2^)] = 0.051	H atoms treated by a mixture of independent and constrained refinement
*wR*(*F*^2^) = 0.142	*w* = 1/[σ^2^(*F*_o_^2^) + (0.0858*P*)^2^] where *P* = (*F*_o_^2^ + 2*F*_c_^2^)/3
*S* = 0.99	(Δ/σ)_max_ < 0.001
3178 reflections	Δρ_max_ = 0.33 e Å^−^^3^
237 parameters	Δρ_min_ = −0.19 e Å^−^^3^
0 restraints	Extinction correction: *SHELXL*, Fc^*^=kFc[1+0.001xFc^2^λ^3^/sin(2θ)]^-1/4^
Primary atom site location: structure-invariant direct methods	Extinction coefficient: 0.0029 (5)

### Special details

**Table:**

Geometry. All e.s.d.'s (except the e.s.d. in the dihedral angle between two l.s. planes) are estimated using the full covariance matrix. The cell e.s.d.'s are taken into account individually in the estimation of e.s.d.'s in distances, angles and torsion angles; correlations between e.s.d.'s in cell parameters are only used when they are defined by crystal symmetry. An approximate (isotropic) treatment of cell e.s.d.'s is used for estimating e.s.d.'s involving l.s. planes.
Refinement. Refinement of *F*^2^ against ALL reflections. The weighted *R*-factor *wR* and goodness of fit *S* are based on *F*^2^, conventional *R*-factors *R* are based on *F*, with *F* set to zero for negative *F*^2^. The threshold expression of *F*^2^ > σ(*F*^2^) is used only for calculating *R*-factors(gt) *etc*. and is not relevant to the choice of reflections for refinement. *R*-factors based on *F*^2^ are statistically about twice as large as those based on *F*, and *R*- factors based on ALL data will be even larger.

### Fractional atomic coordinates and isotropic or equivalent isotropic displacement parameters (Å^2^)

**Table:**

	*x*	*y*	*z*	*U*_iso_*/*U*_eq_	
O1	0.3603 (4)	0.42899 (18)	0.25960 (15)	0.0803 (8)	
N1	0.3795 (4)	0.54279 (16)	0.38661 (17)	0.0502 (6)	
C2	0.2579 (4)	0.48488 (18)	0.41767 (17)	0.0449 (6)	
C3	0.1580 (4)	0.44089 (18)	0.56238 (18)	0.0480 (7)	
H3A	0.2381	0.4495	0.6103	0.058*	
N4	−0.0460 (3)	0.44531 (15)	0.58692 (15)	0.0497 (6)	
C5	−0.1003 (4)	0.53164 (17)	0.57301 (18)	0.0502 (7)	
H5A	−0.0557	0.5667	0.6169	0.060*	
H5B	−0.2378	0.5368	0.5690	0.060*	
C6	−0.0061 (4)	0.55619 (17)	0.49369 (18)	0.0458 (7)	
H6A	0.0052	0.6161	0.4890	0.055*	
H6B	−0.0769	0.5350	0.4475	0.055*	
C7	0.1943 (4)	0.51410 (16)	0.50026 (16)	0.0415 (6)	
C8	0.3434 (4)	0.57872 (15)	0.51991 (17)	0.0429 (6)	
C9	0.3820 (4)	0.62291 (17)	0.58936 (18)	0.0483 (7)	
H9A	0.3178	0.6114	0.6374	0.058*	
C10	0.5188 (5)	0.68503 (18)	0.5862 (2)	0.0548 (8)	
H10A	0.5490	0.7144	0.6330	0.066*	
C11	0.6111 (4)	0.70392 (17)	0.5142 (2)	0.0545 (8)	
H11A	0.7014	0.7463	0.5134	0.065*	
C12	0.5718 (4)	0.66105 (18)	0.4433 (2)	0.0519 (8)	
H12A	0.6323	0.6740	0.3948	0.062*	
C13	0.4380 (4)	0.59779 (17)	0.44827 (19)	0.0451 (6)	
C14	0.2033 (5)	0.35663 (17)	0.5262 (2)	0.0561 (8)	
H14A	0.1569	0.3129	0.5615	0.067*	
H14B	0.3401	0.3502	0.5203	0.067*	
C15	0.1066 (5)	0.35033 (17)	0.4430 (2)	0.0542 (8)	
H15A	0.1239	0.2937	0.4224	0.065*	
C16	0.2053 (4)	0.41012 (19)	0.3855 (2)	0.0507 (7)	
C17	0.2757 (5)	0.3843 (2)	0.3081 (2)	0.0644 (9)	
H17A	0.2557	0.3293	0.2932	0.077*	
C18	−0.2094 (6)	0.3305 (2)	0.3113 (2)	0.0717 (10)	
H18A	−0.3063	0.2909	0.2970	0.108*	
H18B	−0.0862	0.3049	0.3064	0.108*	
H18C	−0.2167	0.3774	0.2755	0.108*	
C19	−0.2388 (5)	0.35864 (19)	0.3968 (2)	0.0591 (8)	
H19A	−0.3632	0.3725	0.4110	0.071*	
C20	−0.1099 (5)	0.36621 (16)	0.4544 (2)	0.0519 (7)	
C21	−0.1658 (4)	0.3855 (2)	0.5405 (2)	0.0578 (8)	
H21A	−0.2951	0.4069	0.5398	0.069*	
H21B	−0.1682	0.3336	0.5703	0.069*	
O2	0.6272 (5)	0.5922 (2)	0.2494 (2)	0.0966 (10)	
C22	0.8184 (10)	0.5554 (3)	0.2572 (3)	0.122 (2)	
H22A	0.8317	0.5300	0.3102	0.146*	
H22B	0.8352	0.5125	0.2165	0.146*	
C23	0.9609 (10)	0.6183 (5)	0.2469 (4)	0.142 (2)	
H23A	1.0852	0.5935	0.2498	0.213*	
H23B	0.9482	0.6591	0.2890	0.213*	
H23C	0.9449	0.6445	0.1951	0.213*	
H1	0.428 (5)	0.544 (2)	0.338 (2)	0.073 (12)*	
H2	0.599 (8)	0.585 (3)	0.193 (3)	0.125 (19)*	

### Atomic displacement parameters (Å^2^)

**Table:**

	*U*^11^	*U*^22^	*U*^33^	*U*^12^	*U*^13^	*U*^23^
O1	0.0735 (16)	0.1134 (19)	0.0539 (16)	−0.0183 (17)	0.0078 (14)	−0.0189 (14)
N1	0.0464 (13)	0.0663 (14)	0.0377 (15)	−0.0053 (12)	0.0064 (12)	0.0009 (11)
C2	0.0343 (13)	0.0568 (14)	0.0435 (17)	0.0022 (13)	−0.0031 (12)	0.0060 (12)
C3	0.0483 (15)	0.0554 (15)	0.0403 (17)	−0.0032 (13)	−0.0033 (13)	0.0104 (12)
N4	0.0471 (13)	0.0575 (13)	0.0446 (15)	−0.0064 (11)	0.0011 (11)	0.0065 (11)
C5	0.0431 (15)	0.0573 (16)	0.0503 (19)	0.0014 (13)	0.0048 (13)	0.0017 (13)
C6	0.0386 (13)	0.0491 (14)	0.0496 (19)	0.0003 (12)	−0.0025 (13)	0.0056 (13)
C7	0.0421 (14)	0.0448 (12)	0.0374 (16)	0.0035 (11)	0.0002 (12)	0.0046 (11)
C8	0.0391 (14)	0.0467 (14)	0.0429 (16)	0.0034 (12)	0.0034 (12)	0.0068 (11)
C9	0.0463 (15)	0.0536 (15)	0.0452 (17)	0.0026 (14)	0.0017 (14)	−0.0012 (12)
C10	0.0530 (18)	0.0528 (16)	0.059 (2)	0.0033 (14)	−0.0081 (16)	−0.0091 (15)
C11	0.0400 (15)	0.0482 (14)	0.075 (2)	−0.0026 (13)	−0.0006 (16)	0.0034 (14)
C12	0.0408 (15)	0.0559 (16)	0.059 (2)	−0.0014 (13)	0.0056 (15)	0.0046 (14)
C13	0.0402 (13)	0.0517 (14)	0.0433 (17)	0.0034 (12)	0.0011 (13)	0.0033 (12)
C14	0.0549 (18)	0.0500 (15)	0.063 (2)	0.0010 (14)	−0.0056 (16)	0.0103 (14)
C15	0.0532 (17)	0.0443 (13)	0.065 (2)	0.0029 (13)	−0.0015 (17)	−0.0026 (13)
C16	0.0426 (14)	0.0571 (16)	0.0523 (18)	0.0017 (13)	−0.0007 (14)	−0.0054 (14)
C17	0.0554 (18)	0.074 (2)	0.063 (2)	−0.0033 (18)	−0.0025 (18)	−0.0231 (17)
C18	0.067 (2)	0.074 (2)	0.074 (3)	−0.0043 (19)	−0.007 (2)	−0.0130 (18)
C19	0.0529 (18)	0.0570 (16)	0.067 (2)	−0.0049 (15)	0.0005 (17)	−0.0034 (15)
C20	0.0522 (17)	0.0448 (14)	0.059 (2)	−0.0074 (13)	0.0003 (16)	0.0029 (13)
C21	0.0508 (17)	0.0622 (17)	0.061 (2)	−0.0128 (14)	0.0042 (16)	0.0074 (15)
O2	0.0799 (19)	0.159 (3)	0.0515 (18)	−0.028 (2)	0.0055 (16)	−0.0156 (18)
C22	0.194 (6)	0.102 (3)	0.068 (3)	−0.011 (4)	−0.040 (4)	0.007 (3)
C23	0.128 (5)	0.167 (6)	0.132 (5)	−0.044 (4)	0.007 (4)	−0.002 (5)

### Geometric parameters (Å, º)

**Table:**

O1—C17	1.228 (4)	C12—C13	1.387 (4)
N1—C2	1.363 (4)	C12—H12A	0.9300
N1—C13	1.409 (4)	C14—C15	1.533 (5)
N1—H1	0.87 (4)	C14—H14A	0.9700
C2—C16	1.365 (4)	C14—H14B	0.9700
C2—C7	1.508 (4)	C15—C16	1.518 (4)
C3—N4	1.489 (4)	C15—C20	1.551 (5)
C3—C14	1.515 (4)	C15—H15A	0.9800
C3—C7	1.582 (3)	C16—C17	1.431 (5)
C3—H3A	0.9800	C17—H17A	0.9300
N4—C5	1.459 (4)	C18—C19	1.496 (5)
N4—C21	1.489 (4)	C18—H18A	0.9600
C5—C6	1.518 (4)	C18—H18B	0.9600
C5—H5A	0.9700	C18—H18C	0.9600
C5—H5B	0.9700	C19—C20	1.317 (5)
C6—C7	1.564 (4)	C19—H19A	0.9300
C6—H6A	0.9700	C20—C21	1.505 (5)
C6—H6B	0.9700	C21—H21A	0.9700
C7—C8	1.509 (4)	C21—H21B	0.9700
C8—C9	1.375 (4)	O2—C22	1.471 (7)
C8—C13	1.389 (4)	O2—H2	0.95 (5)
C9—C10	1.387 (4)	C22—C23	1.433 (8)
C9—H9A	0.9300	C22—H22A	0.9700
C10—C11	1.386 (4)	C22—H22B	0.9700
C10—H10A	0.9300	C23—H23A	0.9600
C11—C12	1.384 (4)	C23—H23B	0.9600
C11—H11A	0.9300	C23—H23C	0.9600
			
C2—N1—C13	109.9 (3)	C8—C13—N1	109.6 (2)
C2—N1—H1	128 (2)	C3—C14—C15	108.6 (2)
C13—N1—H1	122 (2)	C3—C14—H14A	110.0
N1—C2—C16	128.7 (3)	C15—C14—H14A	110.0
N1—C2—C7	108.1 (2)	C3—C14—H14B	110.0
C16—C2—C7	123.0 (3)	C15—C14—H14B	110.0
N4—C3—C14	110.6 (2)	H14A—C14—H14B	108.4
N4—C3—C7	107.2 (2)	C16—C15—C14	108.3 (3)
C14—C3—C7	112.2 (2)	C16—C15—C20	114.7 (2)
N4—C3—H3A	109.0	C14—C15—C20	108.3 (3)
C14—C3—H3A	109.0	C16—C15—H15A	108.4
C7—C3—H3A	109.0	C14—C15—H15A	108.4
C5—N4—C3	104.7 (2)	C20—C15—H15A	108.4
C5—N4—C21	112.8 (2)	C2—C16—C17	120.6 (3)
C3—N4—C21	111.8 (2)	C2—C16—C15	116.1 (3)
N4—C5—C6	105.6 (2)	C17—C16—C15	122.1 (3)
N4—C5—H5A	110.6	O1—C17—C16	125.3 (3)
C6—C5—H5A	110.6	O1—C17—H17A	117.3
N4—C5—H5B	110.6	C16—C17—H17A	117.3
C6—C5—H5B	110.6	C19—C18—H18A	109.5
H5A—C5—H5B	108.7	C19—C18—H18B	109.5
C5—C6—C7	102.6 (2)	H18A—C18—H18B	109.5
C5—C6—H6A	111.2	C19—C18—H18C	109.5
C7—C6—H6A	111.2	H18A—C18—H18C	109.5
C5—C6—H6B	111.2	H18B—C18—H18C	109.5
C7—C6—H6B	111.2	C20—C19—C18	127.8 (3)
H6A—C6—H6B	109.2	C20—C19—H19A	116.1
C2—C7—C8	101.8 (2)	C18—C19—H19A	116.1
C2—C7—C6	109.8 (2)	C19—C20—C21	121.4 (3)
C8—C7—C6	109.8 (2)	C19—C20—C15	124.7 (3)
C2—C7—C3	113.6 (2)	C21—C20—C15	113.7 (3)
C8—C7—C3	119.0 (2)	N4—C21—C20	118.1 (2)
C6—C7—C3	102.9 (2)	N4—C21—H21A	107.8
C9—C8—C13	120.0 (3)	C20—C21—H21A	107.8
C9—C8—C7	132.2 (3)	N4—C21—H21B	107.8
C13—C8—C7	107.5 (2)	C20—C21—H21B	107.8
C8—C9—C10	118.5 (3)	H21A—C21—H21B	107.1
C8—C9—H9A	120.7	C22—O2—H2	103 (3)
C10—C9—H9A	120.7	C23—C22—O2	110.0 (5)
C11—C10—C9	120.9 (3)	C23—C22—H22A	109.7
C11—C10—H10A	119.5	O2—C22—H22A	109.7
C9—C10—H10A	119.5	C23—C22—H22B	109.7
C12—C11—C10	121.3 (3)	O2—C22—H22B	109.7
C12—C11—H11A	119.3	H22A—C22—H22B	108.2
C10—C11—H11A	119.3	C22—C23—H23A	109.5
C11—C12—C13	116.8 (3)	C22—C23—H23B	109.5
C11—C12—H12A	121.6	H23A—C23—H23B	109.5
C13—C12—H12A	121.6	C22—C23—H23C	109.5
C12—C13—C8	122.4 (3)	H23A—C23—H23C	109.5
C12—C13—N1	128.0 (3)	H23B—C23—H23C	109.5
			
C13—N1—C2—C16	−162.4 (3)	C10—C11—C12—C13	−0.9 (4)
C13—N1—C2—C7	13.9 (3)	C11—C12—C13—C8	1.4 (4)
C14—C3—N4—C5	−146.6 (2)	C11—C12—C13—N1	−176.8 (3)
C7—C3—N4—C5	−24.0 (3)	C9—C8—C13—C12	−0.4 (4)
C14—C3—N4—C21	−24.2 (3)	C7—C8—C13—C12	173.6 (2)
C7—C3—N4—C21	98.4 (3)	C9—C8—C13—N1	178.1 (2)
C3—N4—C5—C6	40.5 (3)	C7—C8—C13—N1	−7.9 (3)
C21—N4—C5—C6	−81.3 (3)	C2—N1—C13—C12	174.5 (3)
N4—C5—C6—C7	−40.3 (3)	C2—N1—C13—C8	−3.9 (3)
N1—C2—C7—C8	−17.6 (3)	N4—C3—C14—C15	70.8 (3)
C16—C2—C7—C8	159.0 (3)	C7—C3—C14—C15	−48.7 (3)
N1—C2—C7—C6	98.7 (3)	C3—C14—C15—C16	68.8 (3)
C16—C2—C7—C6	−84.7 (3)	C3—C14—C15—C20	−56.2 (3)
N1—C2—C7—C3	−146.8 (2)	N1—C2—C16—C17	−1.8 (5)
C16—C2—C7—C3	29.8 (4)	C7—C2—C16—C17	−177.7 (3)
C5—C6—C7—C2	145.0 (2)	N1—C2—C16—C15	165.5 (3)
C5—C6—C7—C8	−104.0 (2)	C7—C2—C16—C15	−10.3 (4)
C5—C6—C7—C3	23.7 (3)	C14—C15—C16—C2	−38.9 (4)
N4—C3—C7—C2	−119.1 (3)	C20—C15—C16—C2	82.2 (3)
C14—C3—C7—C2	2.4 (3)	C14—C15—C16—C17	128.2 (3)
N4—C3—C7—C8	121.1 (3)	C20—C15—C16—C17	−110.7 (3)
C14—C3—C7—C8	−117.4 (3)	C2—C16—C17—O1	−11.7 (5)
N4—C3—C7—C6	−0.6 (3)	C15—C16—C17—O1	−178.3 (3)
C14—C3—C7—C6	120.9 (3)	C18—C19—C20—C21	−172.5 (3)
C2—C7—C8—C9	−171.9 (3)	C18—C19—C20—C15	2.8 (5)
C6—C7—C8—C9	71.9 (4)	C16—C15—C20—C19	64.9 (4)
C3—C7—C8—C9	−46.2 (4)	C14—C15—C20—C19	−173.9 (3)
C2—C7—C8—C13	15.1 (3)	C16—C15—C20—C21	−119.4 (3)
C6—C7—C8—C13	−101.1 (3)	C14—C15—C20—C21	1.7 (3)
C3—C7—C8—C13	140.8 (3)	C5—N4—C21—C20	85.3 (3)
C13—C8—C9—C10	−1.2 (4)	C3—N4—C21—C20	−32.3 (4)
C7—C8—C9—C10	−173.5 (3)	C19—C20—C21—N4	−139.5 (3)
C8—C9—C10—C11	1.8 (4)	C15—C20—C21—N4	44.7 (4)
C9—C10—C11—C12	−0.7 (5)		

### Hydrogen-bond geometry (Å, º)

**Table:**

*D*—H···*A*	*D*—H	H···*A*	*D*···*A*	*D*—H···*A*
N1—H1···O1	0.87 (3)	2.31 (3)	2.785 (4)	115 (3)
N1—H1···O2	0.87 (3)	2.17 (3)	2.961 (4)	152 (3)
O2—H2···N4^i^	0.96 (5)	1.85 (5)	2.805 (4)	172 (5)

Symmetry code: (i) −*x*+1/2, −*y*+1, *z*−1/2.
